# Developing a National eHealth Enterprise Architecture in Botswana: a digital health case study

**DOI:** 10.1093/oodh/oqag008

**Published:** 2026-04-28

**Authors:** Audrey Masizana, Mwariri Mwangi, Rugo Kisanga, Kagiso Ndlovu, Badisa Mosesane, Michelle Moghadassi, Fitti Weissglas, Sinka Matengu, Tony Chebani

**Affiliations:** Department of Computer Science, University of Botswana, Gaborone, South-East District, Botswana; Global Health Programs, University of California, San Francisco, Nairobi, Nairobi City County, Kenya; Global Health Programs, University of California, San Francisco, Nairobi, Nairobi City County, Kenya; Sir Ketumile Masire Teaching Hospital, University of Botswana, Gaborone, South-East District, Botswana; Department of Computer Science, University of Botswana, Gaborone, South-East District, Botswana; Institute of Global Health Sciences, University of California, San Francisco, Nairobi, Nairobi City County, Kenya; Institute of Global Health Sciences, University of California, San Francisco, Nairobi, Nairobi City County, Kenya; Department of Health Informatics, Ministry of Health, Gaborone, South-East District, Botswana; Department of Health Informatics, Ministry of Health, Gaborone, South-East District, Botswana

**Keywords:** eHealth, interoperability, enterprise architecture, TOGAF, OpenHIE, Botswana

## Abstract

Botswana’s health sector comprises numerous digital health systems that operate in isolation, thereby constraining interoperability, limiting data exchange, and hindering coordinated patient care. Although national strategies such as the Botswana eHealth Strategy (2020–24) articulate overarching digital health objectives, they offer limited technical direction for harmonizing digital investments and achieving system-wide interoperability. This article presents a national digital health case study detailing the development of the Botswana eHealth Enterprise Architecture, a strategic blueprint intended to guide the integration and governance of digital health systems nationwide. The architecture was formulated through a participatory process involving stakeholders from the Ministry of Health, healthcare facilities, academic institutions, and international partners. The initiative was informed by internationally recognized frameworks, including The Open Group Architecture Framework and the Open Health Information Exchange interoperability model. Through an iterative development process undertaken between 2023 and 2024, the architecture delineated four core layers—business, data, application, and technology—and established a national interoperability framework grounded in open standards, including HL7 Fast Healthcare Interoperability Resources and HL7 v2. The resulting architecture further introduced a suite of shared national digital health services, such as a Master Patient Index, a Shared Health Record, and national facility and provider registries. Key lessons from Botswana’s experience underscore the importance of robust governance structures, inclusive stakeholder engagement, the adoption of open standards, and phased implementation approaches in the development of national digital health architectures. This case study offers practical insights for other low- and middle-income countries seeking to mitigate fragmentation in their health information systems and to implement interoperable digital health ecosystems.

## Introduction

This case study documents the process undertaken to develop the Botswana eHealth Enterprise Architecture between 2023 and 2024. The initiative was led by the Ministry of Health, with support from academic and international partners, and involved extensive engagement with stakeholders across both the public and private health sectors.

Digital transformation is increasingly recognized as a critical enabler of improved health system performance, strengthened service delivery, and enhanced evidence-based decision-making. Nevertheless, many low- and middle-income countries (LMICs) continue to face challenges associated with fragmented health information ecosystems, characterized by multiple standalone systems, inconsistent data standards, and limited interoperability. These constraints often result in duplicative data entry, incomplete patient records, and inefficient information flows across healthcare facilities.

Enterprise architecture (EA) has emerged globally as a strategic approach to addressing fragmentation within digital health ecosystems. It provides a structured methodology for aligning business processes, data governance, applications, and technology infrastructure within a coherent national digital health framework. Internationally recognized frameworks—such as the World Health Organization’s Digital Health Platform guidance (2020), (The Open Group Architecture Framework (TOGAF), 1999) updated ((The Open Group Architecture Framework (TOGAF), 2022), and the Open Health Information Exchange (OpenHIE) architecture (2013)—have been widely adopted to support scalable, standards-based health information exchange and enterprise digital health platforms.

Similar case studies have also been documented across the African continent and other low- and middle-income countries (LMICs). Mwebaze (2023) examines Uganda’s digital health policy environment and highlights key interventions within national systems. The study identifies several supply-side challenges, including weak governance structures, inadequate funding, limited infrastructure, a shortage of skilled personnel, and low levels of digital health literacy. Despite these constraints, the author underscores the significant potential of mobile health (mHealth) technologies to strengthen public health programmes.

In their analysis of EA in LMICs, Mudaly et al. (2013) conclude that these settings present complex environments for the development of national health information systems, necessitating rigorous and well-structured approaches. Similarly, Higman et al. (2019) emphasize the importance of interoperability, system flexibility, and the adoption of open group models, data standards, and software.

Furthermore, Adenuga et al. (2015) propose an eHealth architectural model for South Africa aimed at addressing integration and interoperability challenges within the country’s health sector systems.

## Botswana context

In Botswana, similar challenges persist, with numerous programme-specific digital systems operating alongside paper-based registers and legacy applications. This has resulted in fragmented data repositories and limited integration of patient information across the health sector. Recognizing the potential of digital technologies to strengthen healthcare delivery, Botswana has established a supportive policy environment that includes the Botswana National Health Policy (2011), Botswana Integrated Health Service Plan (2010), Botswana Data Protection Act (2018), The Botswana eHealth Strategy (2020–24), as well as the Botswana Health Data Collaborative Roadmap (2020).

These frameworks emphasize the importance of digital health in improving health information management and enabling data-driven decision-making. However, while they articulate national priorities, they provide limited technical guidance for coordinating digital investments across systems, standardizing data exchange, and ensuring interoperability among health information systems.

In response to these challenges, Botswana initiated the development of the Botswana eHealth Enterprise Architecture (BeHEA) as a national blueprint to guide digital health investments and strengthen interoperability across the health sector.

The objective of this case study is to describe and distil transferable lessons from the development of Botswana’s national eHealth Enterprise Architecture, with particular emphasis on governance, interoperability design, and phased implementation within an LMIC country context. By documenting Botswana’s experience, this study contributes to the growing body of literature on national digital health EAs and offers insights that may inform similar initiatives in other LMICs.

## Governance and decision-making

The development was led by the Ministry of Health, which provided strategic direction and ensured that the EA framework aligned with the health sector’s long-term objectives. In this leadership role, the Ministry was responsible for defining the vision, establishing guiding principles, and prioritizing architectural initiatives.

The Enterprise Architecture Development Team, tasked with designing and developing the EA, worked in close collaboration with Ministry leadership. The team comprised enterprise architects, business analysts, IT architects, health subject-matter experts, and stakeholder representatives. The Ministry of Health, through its Health Informatics Unit, served as the custodian of the process and was responsible for validating the overall development.

In addition, a resolute stakeholder engagement team was established to support the initiative. This team included representatives from the Ministry of Health and its affiliated sectors, ensuring broad-based participation and alignment throughout the architecture development approach.

## Stakeholder engagement and initial scoping

The BeHEA project commenced with a stakeholder mapping exercise to identify key actors across the health sector, including the Ministry of Health, academic partners, international organizations, and other sector stakeholders. Local developers, frontline health workers, and health informatics specialists also played a critical role in shaping the EA, ensuring that the resulting blueprint was aligned with operational realities and on-the-ground requirements.

A project kick-off meeting established a shared vision for the EA, along with clearly defined objectives and scope, ensuring alignment with national strategic priorities from the outset. This was followed by a series of workshops through which the project team gathered domain knowledge on existing health system bottlenecks and opportunities for improvement. Emerging cross-cutting themes were identified and subsequently informed follow-up interview sessions with key informants.

The architecture design evolved iteratively over roughly a year (late 2023–24), organized into five phases:

1. **Planning and Visioning.** Initial stakeholder consultations were used to define the vision, scope, and guiding principles of EA. These discussions emphasized interoperability, reduction of system duplication, and alignment with national health priorities. The output of this phase was a synthesized view of the business requirements across the sector.

2. **Baseline Architecture Assessment**. The project team assessed the existing digital health landscape in Botswana. This involved mapping current information systems, identifying data flows, and documenting existing infrastructure and interoperability challenges.

3. **Target Architecture Design.** Based on insights from the baseline assessment, the team developed a target architecture, describing the desired future state of Botswana’s digital health ecosystem. The design incorporated business, data, application, and technology architecture layers.

4. **Development of the Interoperability Framework**. An interoperability framework was developed to enable standardized data exchange across systems. Botswana adopted an architecture aligned with the OpenHIE Community (2013), as it offers a modular, standards-based approach for integrating multiple digital health systems at a national scale. The OpenHIE model promotes the use of shared interoperability services—such as client registries, facility registries, and shared health records (SHRs)—connected through a central interoperability layer.

The framework incorporated international interoperability standards, including HL7 v2 for messaging, HL7 Fast Healthcare Interoperability Resources (FHIR) for clinical data exchange, and proposed the use of OpenHIM (Open Health Information Mediator) as the national interoperability middleware. OpenHIM was selected due to its strong alignment with the OpenHIE reference architecture and its design specifically for national digital health ecosystems. Unlike general-purpose integration engines, OpenHIM functions as a centralized interoperability layer that supports routing, transformation, orchestration, and monitoring of health data transactions between systems.

In addition, it provides built-in governance capabilities such as transaction logging, audit trails, authentication, and access control, enabling the Ministry of Health to effectively monitor and manage data exchange across the national digital health environment. Its open-source model, coupled with an active global implementation community, further enhances its suitability for LMICs. For Botswana, the adoption of OpenHIM therefore represents a practical balance between technical capability, sustainability, and alignment with internationally recognized digital health interoperability frameworks.

The project team also reviewed key national policies, existing EA documentation, and case studies from countries that had faced similar interoperability challenges. Several African countries have adopted interoperability approaches informed by the OpenHIE to integrate diverse digital health systems and reduce fragmentation, although levels of implementation vary considerably.

Countries such as Tanzania (Tanzania Ministry of Health, Community Development, Gender, Elderly and Children–Tanzania (MoHCDGEC) 2020, Nsaghurwe et al. 2021) and Rwanda (Crichton et al. 2013, Nzarama et al. 2024) have established national health information exchanges that connect electronic medical record systems, laboratory systems, and national platforms. Kenya (Kenya Ministry of Heath, 2020) has implemented a national interoperability framework linking digital health applications and reporting systems such as the District Health Information Software 2 (DHIS2) (Thaiya et al. 2021). Nigeria (Dalhatu et al. 2023), along with South Africa (National Department of Health–South Africa 2019), Malawi, Ethiopia, Uganda, and Mozambique are at various stages of developing or piloting similar infrastructures.

Collectively, these initiatives reflect a broader regional shift towards modular, standards-based architectures that rely on shared registries, interoperability middleware, and standardized APIs to enable data exchange and national-level data aggregation for monitoring and decision-making. Governance structures differ across contexts, with Rwanda and Ethiopia adopting more centralized models, Kenya and Nigeria operating within more decentralized systems, and Malawi and Tanzania combining strong ministry leadership with substantial donor support. In addition, the Africa CDC African Union Health Information Exchange Guidelines and Standards (Adane et al. 2022) were also consulted.

These sources underscored both the importance of early stakeholder governance and the value of clearly defined and consistently applied data standards.

5. **Validation and Governance**. Draft versions of the architecture were reviewed through stakeholder workshops and consultations. Feedback was systematically incorporated into the final architecture blueprint to ensure both policy alignment and practical feasibility. The final version was subsequently endorsed through the Ministry of Health’s established governance structures.

Throughout these phases, the team applied a combination of desk research, participatory engagement, and pilot testing of selected interoperability use cases. More than 7 workshops were conducted, each involving over 100 participants drawn from various parts of the health sector. Participants were formally nominated to represent relevant departmental perspectives and included business leaders, IT leaders, subject-matter experts, and end users.

The significant effort invested in coordinating broad stakeholder participation ultimately strengthened the acceptability and realism of the final blueprint. Although the participatory process required substantial coordination and time, it ensured that the architecture reflected stakeholder needs and fostered broad ownership across the health sector.

In addition, various data collection instruments, including structured interviews, were developed for targeted engagement with over 30 individuals across different domains. These platforms served as observational and consultative data collection mechanisms, generating synthesized inputs that informed the development of a shared vision, roadmap priorities, roles and responsibilities, governance structures, and feedback mechanisms.

## Architecture outputs

The Open Group Architecture Framework (The Open Group 1999) informed the development of the BeHEA. This framework offered a structured, multi-phase development process spanning business, data, application, and technology layers. Its adaptability made it suitable for Botswana.

### Business architecture

In developing the business requirements, the Blueprint followed (World Health Organisation (WHO), 2021) health system building blocks framework of 2010 which provides a structured approach for describing the sector across the public and private institutions, identified through six core components as articulated in Gavi Global Alliance Civil Societies Project (GAVI Civil Society Organizations (CSOs) 2013).

The BeHEA business architecture aligns digital health investments with Botswana’s broader health objectives, ensuring a direct linkage between technology initiatives and improvements in service delivery. A clear delineation of stakeholder roles helps prevent duplication, as each agency or programme has a well-defined understanding of its responsibilities and its contribution to the national digital ecosystem.

The standardization of clinical and administrative processes across health facilities not only streamlines workflows but also reduces redundancy. For example, a unified patient registration process supported by digital tools can minimize repeated data capture. This harmonization contributes to a more coherent system that is easier to manage at both local and national levels, ultimately supporting more timely and accurate decision-making.

**Figure 1:**
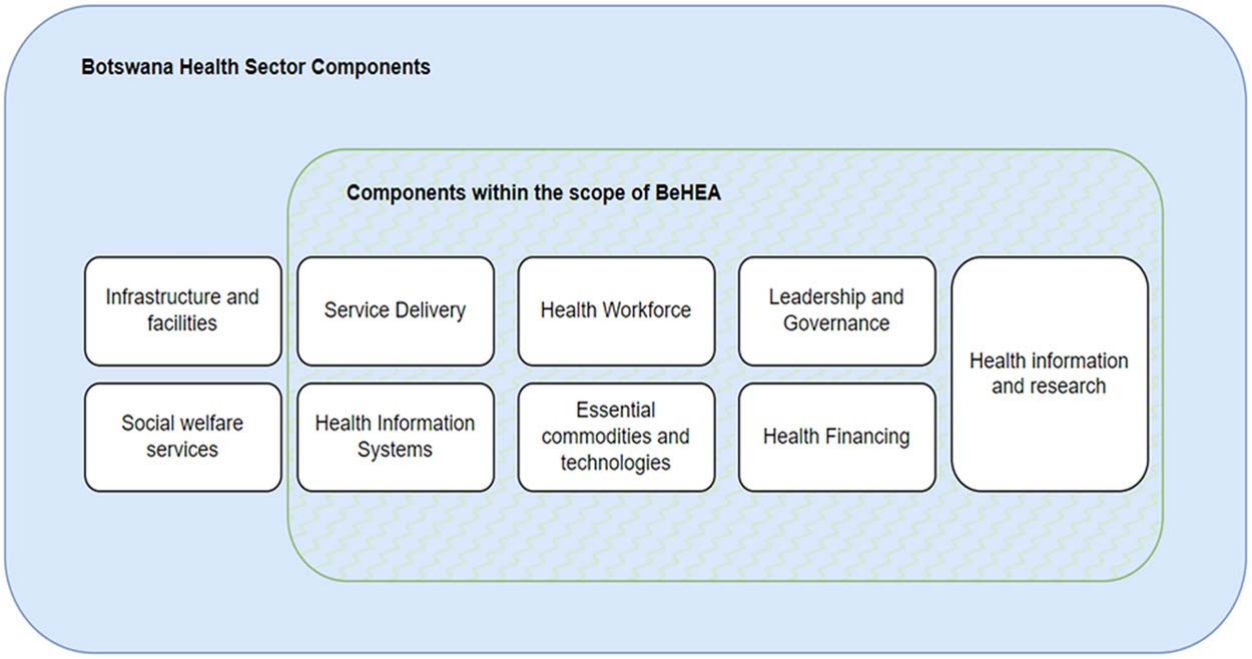
Botswana health sector view of the adapted WHO components.

This adaptation resulted in the identification of seven core areas within the Botswana context, namely: Service Delivery, Health Workforce, Health Information Systems, Medical Products, Financing, Leadership, and Governance, as illustrated in Fig. 1.

BeHEA’s data architecture establishes a structured framework for defining, managing, and sharing health data across the sector. A key strength of BeHEA is its comprehensive interoperability framework, which defines both the technical and governance dimensions of data exchange. It mandates the use of HL7 FHIR for modern applications, while retaining HL7 v2 support for legacy systems. This pragmatic dual-standard approach allows the existing Integrated Patient Management System to continue operating without immediate major modifications, while also providing a clear migration pathway towards FHIR.

Central to this architecture is a common data dictionary that standardizes key data elements such as patient identifiers, facility codes, and diagnoses. In addition, a designated Single Source of Truth model ensures that specific repositories serve as authoritative data sources. For example, the SHR is defined as the national repository for longitudinal patient data, while the Health Facility Registry (HFR) maintains validated information on all health facilities. This approach reduces data fragmentation and promotes consistency across systems.

Governance is embedded within the architecture through clearly defined protocols for data sharing, patient consent, and privacy, ensuring alignment with Botswana’s regulatory framework and international best practices. Security controls, including encryption and audit mechanisms, safeguard patient confidentiality and support the secure, reliable, and controlled data exchange across the health sector.

### Application architecture

The application architecture centres on the digital applications needed for data collection and service delivery, emphasizing interoperability and user-centred design. Where existing solutions are proven—such as DHIS2 for aggregate reporting or OpenMRS for Electorinic Medical Records (EMRs)—BeHEA recommends harnessing and adapting these platforms, minimizing duplication. Shared services (e.g. single sign-on, messaging, analytics) are abstracted to avoid repeated development. This consolidation enhances both cost-effectiveness and uniformity—health workers can expect consistent interfaces for tasks like patient searches or laboratory result entry across different systems. By advocating open standards and standard APIs, the architecture encourages constructive interaction between applications, reducing the previous proliferation of siloed systems.

The blueprint pinpoints **priority systems** for integration: the Botswana EMR, the HFR, national Laboratory Information System (LIS), and the SHR. When new facilities are registered in the HFR, e.g. that information automatically propagates to other connected systems, reducing manual data entry. Likewise, a newly completed lab test from the LIS flows to the SHR and the relevant EMR, ensuring clinicians anywhere in the network can access the result. By outlining these interactions, BeHEA demonstrates how individual systems should interoperate within a cohesive national framework. The result is a more holistic patient record, improved resource tracking, and quicker insight generation for public health decision-making.

**Figure 2:**
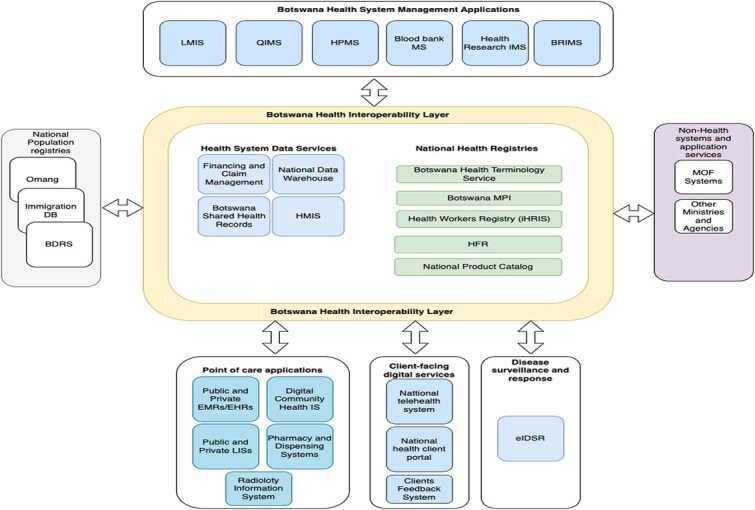
BeHEA application architecture.

Instead of multiple point-to-point connections, each system integrates only once—with OpenHIM mediating and transforming data as necessary as shown in Fig. 2. For instance, a lab system sends HL7 v2 messages to OpenHIM, which can convert them to FHIR if needed and forward them to the SHR or an EMR. This central broker approach dramatically reduces complexity and eases upgrades: if a new data standard emerges, the mediator can be updated without requiring every individual system to retrofit.

The blueprint also prescribes how errors and data duplication are managed, with the Master Patient Index ensuring unique patient identifiers, while the SHR synchronizes clinical records. Detailed security protocols, including encryption and user authentication, reinforce privacy protections and align with Botswana’s legal context (e.g. data protection mandates).

### Technology architecture

BeHEA’s technology architecture specifies infrastructure requirements—hardware, networking, security, and core software platforms. Scalability is paramount: as usage grows, the systems must manage expanded data volumes and user loads without fundamentally overhauling infrastructure. For example, cloud-based platforms are recommended where feasible to ensure elasticity and broad availability, though the architecture also accommodates offline or hybrid setups crucial for remote clinics. Critical security controls—encryption (in transit and at rest), security system protection, and regular backups—are identified to preserve data integrity and user trust. Solutions must align with recognized security frameworks (e.g. ISO 27001). This domain also includes guidelines for procuring hardware or adopting new software, ensuring conformance to the interoperability and security standards mandated by the national blueprint.

## Discussion

### Implications for digital health in Botswana

BeHEA represents a significant step towards addressing the fragmentation of digital health systems in Botswana by establishing a national architectural framework to guide interoperability and integration. The architecture provides a foundation for linking previously siloed digital health applications and improving data exchange across systems. However, while the framework has been defined, many of the anticipated benefits—such as seamless access to patient referrals, laboratory results, and medication histories across facilities—remain dependent on the progressive implementation and scaling of interoperable systems. If fully implemented, integrated data could strengthen health system management by enabling more timely visibility of operational indicators and supporting faster responses to disease outbreaks or supply shortages.

At the same time, translating architectural design into practical implementation requires substantial organizational change. Evidence from digital health implementations indicates that factors such as workforce capacity, workflow redesign, and user acceptance often determine the success or failure of health information systems. As such, health worker training, sustained stakeholder engagement, and strong governance mechanisms are critical to supporting effective adoption.

While the architecture also creates opportunities for telemedicine, advanced analytics, and artificial intelligence (AI)-supported decision-making, these innovations remain aspirational at this stage. Their realization will depend on sustained investment, improved data quality, and continued strengthening of institutional capacity.

### Interoperability challenges and lessons learned

Developing BeHEA highlighted several critical lessons. First, stakeholder alignment is essential but time-consuming, requiring inclusive engagement to reconcile differing programme priorities, departmental needs, and public–private interests. Shared decision-making fosters co-ownership and ensures that the architecture reflects a common vision.

Second, legacy systems present significant technical challenges. Bridging older platforms using standards-based solutions (e.g. HL7 v2–FHIR via OpenHIM) can sustain interoperability while enabling a gradual and managed transition towards modern systems.

Third, resource constraints make open-source tools particularly valuable, as they reduce costs and provide access to global implementation communities and support networks.

Finally, the experience reinforces that technology alone is insufficient. Robust governance structures, clear data-sharing agreements, and sustained institutional commitment are essential for achieving and maintaining interoperability. Iterative feedback, the use of established global frameworks, and adequate time for consultation are key determinants of successful design and adoption.

## Future directions

Future implementation of BeHEA will follow a phased pilot approach, integrating key health information systems such as laboratory, pharmacy, and logistics platforms. This will allow technical and workflow-related challenges to be identified and addressed before a nationwide scale-up.

Strengthening local capacity is a key priority, with targeted training for health IT personnel on standards such as FHIR, HL7, OpenHIM, and EA governance.

The Ministry of Health also plans to establish a dedicated eHealth Architecture Unit to oversee compliance, coordinate expansion, and ensure alignment with emerging digital health solutions.

As a living framework, BeHEA is expected to evolve in line with national health priorities. Future directions include the comprehensive integration of telemedicine, enabling linkage between remote consultations and the SHR, as well as the gradual adoption of advanced analytics and AI to support clinical decision-making, particularly for chronic disease management.

Cultural adaptation and organizational change management will be essential to ensure successful adoption. The initiative also presents important opportunities for further research in areas such as interoperability, data governance, and health data warehousing.

## Conclusion

The BeHEA demonstrates how a structured, stakeholder-driven approach can transform digital health within an LMIC context. By combining global frameworks such as TOGAF and OpenHIE with Botswana’s national policy environment, BeHEA addresses long-standing fragmentation in health information systems through a central interoperability layer, standardized data protocols, and shared registries that ensure clinical and administrative information is accurate, secure, and accessible.

BeHEA provides a unified vision for digital health investments, reducing duplication and optimizing limited resources. For frontline health workers, it enables more streamlined workflows and faster access to patient information, while patients benefit from improved continuity of care, reduced waiting times, and expanded service delivery modalities such as telemedicine. Lessons from stakeholder engagement, legacy system integration, and governance processes highlight the importance of inclusive planning and iterative refinement in achieving successful adoption.

The flexible architecture supports phased expansion, including telemedicine integration, advanced analytics, and AI-enabled decision support, while remaining responsive to evolving national health priorities. Its long-term success will depend on sustained capacity building, cultural and organizational adaptation, and strong governance mechanisms. Importantly, BeHEA provides a model for other LMICs seeking to implement interoperable, standards-based digital health ecosystems, demonstrating how global frameworks can be effectively adapted to local contexts to achieve sustainable, scalable, and patient-centred health system transformation.

Looking ahead, BeHEA is well aligned with Botswana’s goal of modernizing primary healthcare by 2030. It offers a national coordination framework for introducing new digital services without creating additional data silos.

## Data Availability

The data that support the findings of this study may be provided with permission from Ministry of Health, Botswana in line with the ethics approval process. Not all data may be available subject to funding protocols.
